# Association of rare variants in *ARSA* with Parkinson’s disease

**DOI:** 10.1101/2023.03.08.23286773

**Published:** 2023-03-13

**Authors:** Konstantin Senkevich, Mariia Beletskaia, Aliza Dworkind, Eric Yu, Jamil Ahmad, Jennifer A. Ruskey, Farnaz Asayesh, Dan Spiegelman, Stanley Fahn, Cheryl Waters, Oury Monchi, Yves Dauvilliers, Nicolas Dupré, Lior Greenbaum, Sharon Hassin-Baer, Ilya Nagornov, Alexandr Tyurin, Irina Miliukhina, Alla Timofeeva, Anton Emelyanov, Ekaterina Zakharova, Roy N. Alcalay, Sofya Pchelina, Ziv Gan-Or

**Affiliations:** 1. The Neuro (Montreal Neurological Institute-Hospital), McGill University, Montreal, Quebec, Canada; 2. Department of Neurology and neurosurgery, McGill University, Montréal, QC, Canada, Canada; 3. First Pavlov State Medical University of St. Petersburg, Saint-Petersburg, Russia; 4. Department of Physiology, McGill University, Montréal, QC, Canada; 5. Department of Human Genetics, McGill University, Montréal, QC, Canada; 6. Department of Neurology, College of Physicians and Surgeons, Columbia University Medical Center, NY, USA; 7. Department of Clinical Neurosciences and Department of Radiology, University of Calgary, Calgary, Alberta, Canada; 8. Hotchkiss Brain Institute, Cumming School of Medicine, Calgary, Alberta, T2N 4N1 Canada; 9. National Reference Center for Narcolepsy, Sleep Unit, Department of Neurology, Guide-Chauliac Hospital, CHU Montpellier, University of Montpellier, Montpellier, France.; 10. Division of Neurosciences, CHU de Québec, Université Laval, Quebec City, Quebec, Canada; 11. Department of Medicine, Faculty of Medicine, Université Laval, Québec, Canada; 12. The Danek Gertner Institute of Human Genetics, Sheba Medical Center, Tel Hashomer, Israel; 13. The Joseph Sagol Neuroscience Center, Sheba Medical Center, Tel Hashomer, Israel; 14. Sackler Faculty of Medicine, Tel Aviv University, Tel Aviv, Israel; 15. The Movement Disorders Institute, Department of Neurology, Sheba Medical Center, Tel Hashomer, Israel; 16. Research Centre for Medical Genetics, Moscow, Russia.; 17. Institute of the Human Brain of RAS, St. Petersburg, Russia.; 18. Division of Movement Disorders, Tel Aviv Sourasky Medical Center; Tel Aviv, Israel.

**Keywords:** Lysosomal genes, Parkinson’s disease, *ARSA*, rare variants

## Abstract

**Background:**

Several lysosomal genes are associated with Parkinson’s disease (PD), yet the association between PD and *ARSA*, which encodes for the enzyme arylsulfatase A, remains controversial.

**Objectives:**

To evaluate the association between rare *ARSA* variants and PD.

**Methods:**

To study possible association of rare variants (minor allele frequency<0.01) in *ARSA* with PD, we performed burden analyses in six independent cohorts with a total of 5,801 PD patients and 20,475 controls, using optimized sequence Kernel association test (SKAT-O), followed by a meta-analysis.

**Results:**

We found evidence for an association between functional *ARSA* variants and PD in four independent cohorts (P≤0.05 in each) and in the meta-analysis (P=0.042). We also found an association between loss-of-function variants and PD in the UKBB cohort (P=0.005) and in the meta-analysis (P=0.049). However, despite replicating in four independent cohorts, these results should be interpreted with caution as no association survived correction for multiple comparisons. Additionally, we describe two families with potential co-segregation of the *ARSA* variant p.E384K and PD.

**Conclusions:**

Rare functional and loss-of-function *ARSA* variants may be associated with PD. Further replication in large case-control cohorts and in familial studies is required to confirm these associations.

## Introduction

**Figure 1.**
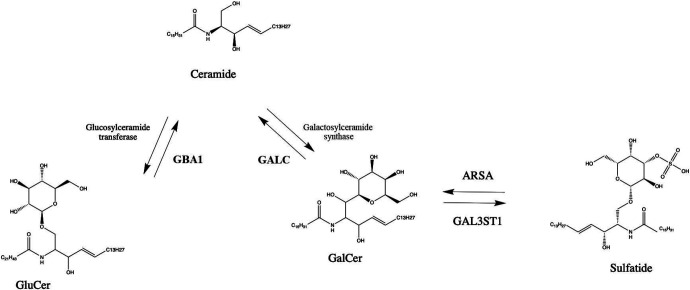
The role of ARSA and GBA1 in sphingolipid metabolism. GluCer- glucosylceramide; GalCer- galactosyleramide; ARSA- arylsulfatase A; GALC- galactosylceramidase; GBA1- galactosylceramidase

Lysosomal genes play a prominent role in the pathogenesis of Parkinson’s disease (PD).^1^ Variants in *GBA1* are amongst the most important risk factors of PD,^2^ and mutations in other lysosomal storage disorder genes have also been associated with PD (e.g. *ASAH1, GALC, SMPD1*).^3-7^ Homozygous or compound heterozygous mutations in *ARSA* may lead to the autosomal recessive lysosomal storage disorder metachromatic leukodystrophy (MLD).^8^ Located on chromosome 22q13.33, the *ARSA* gene encodes arylsulfatase A, which hydrolyzes sulfatides to galactosylceramide and sulfate^8^ (Figure 1). Consequently, hydrolysis of galactosylceramide occurs by the lysosomal enzyme galactosylceramidase, encoded by *GALC*, which is nominated as a PD gene by genome-wide association studies and targeted analyses.^6, 7, 9^

The genetic association between *ARSA* variants and PD remains controversial.^10-14^ Co-segregation of pathogenic *ARSA* variant was reported in one family with two PD patients, and two studies suggested potential association between rare *ARSA* loss-of-function variants and PD.^10, 12^ In the current study, we aimed to evaluate the association between rare *ARSA* variants and PD in six cohorts of 5,801 PD patients and 20,475 controls and in two families with MLD and PD.

## Methods

### Population

The study population included a total of 5,801 PD patients and 20,475 controls from six cohorts (detailed in Supplementary Table 1). Four cohorts have been collected and sequenced at McGill University: McGill (Quebec, Canada and Montpellier, France), Columbia University (the SPOT study, New York, NY), Sheba Medical Center (Israel) and Pavlov First State Medical university and Institute of Human Brain (Pavlov and Human Brain cohort; Saint-Petersburg, Russia). Additionally, we analyzed data from the UK Biobank (UKBB) and Accelerating Medicines Partnership – Parkinson Disease (AMP-PD) initiatives. The McGill university cohort was recruited in Québec, Canada (partially through the Quebec Parkinson Network, QPN)^15^ and in France. The Columbia cohort was collected in NY and is of mixed ancestry (European, Ashkenazi Jews [AJ] and a minority of Hispanics and Blacks, described in detail previously)^16^. The Sheba cohort, recruited in Israel, includes only participants with full AJ ancestry (by report). Pavlov and Human Brain cohort, recruited in Russia, consist predominantly of patients of European ancestry. All PD patients in these cohorts were diagnosed by movement disorder specialists according to the UK brain bank criteria^17^ or the MDS clinical diagnostic criteria.^18^ The Accelerating Medicines Partnership – Parkinson Disease (AMP-PD, 2.5 release) initiative cohorts were accessed using the Terra platform (https://amp-pd.org/; AMP-PD cohorts detailed in Acknowledgments). The UKBB cohort was accessed using Neurohub (https://www.mcgill.ca/hbhl/neurohub).

We contacted 21 families with MLD (homozygous or compound heterozygous carriers of pathogenic *ARSA* variants) or their representatives through Russian Society of Rare (Orphan) Diseases and sent them out questionnaire to detect family history of PD. We analyzed *ARSA* mutations using sanger sequencing in two selected families with positive PD history to attempt detection of co-segregation of pathogenic variants within PD patients.

All participants signed informed consent forms before entering the studies and study protocols were approved by the institutional review boards.

### Targeted next generation sequencing

The *ARSA* gene was sequenced in the four cohorts collected at McGill University with targeted next generation sequencing by molecular inversion probes (MIPs) as previously described.^19^ All MIPs that were used to sequence *ARSA* are provided (Supplementary Table 2) and the full protocol is available at https://github.com/gan-orlab/MIP_protocol. The library was sequenced using Illumina NovaSeq 6000 SP PE100 platform at the Genome Quebec Innovation Centre. Alignment was performed with Burrows-Wheeler Aligner (hg19)^20^ and Genome Analysis Toolkit (GATK, v3.8) was used for post-alignment quality control and variant calling.^21^ We performed quality control by filtering out variants and samples with reduced quality, using the PLINK software v1.9. SNPs were excluded from analysis if missingness was more than 10%. Variants with a minor allele frequency (MAF) less than 1% and with a minimum quality score (GQ) of 30 were included in the analyses and analyzed at minimal depths of coverage 30x.

### Data quality control and analysis in AMP-PD and UKBB

Quality control procedures of whole genome sequencing for AMP-PD cohorts were performed on individual and variant levels as described by AMP-PD (https://amp-pd.org/whole-genome-data and detailed elsewhere).^22^ Quality control of UKBB whole exome sequencing data was performed using Genome Analysis Toolkit (GATK, v3.8) with minimum depth of coverage 10x and GQ 20 as described previously^23^ and we removed all multi-allelic sites.

Alignment of AMP-PD and UKBB data was performed using the human reference genome (hg38) and coordinates for the *ARSA* gene extraction were chr22:50,622,754-50,628,152. We performed additional filtration procedures using the UKBB and AMP-PD cohorts to exclude non-European individuals (UKB field 21000) and filtered by relatedness to remove any first and second-degree relatives.

#### Annotations and statistical analysis

To functionally annotate genetic variants in all cohorts, we utilized ANNOVAR.^24^ Data on variant pathogenicity were predicted using Combined Annotation Dependent Depletion (CADD) score and Varsome.^25, 26^ To analyze rare variants (MAF<0.01), an optimized sequence Kernel association test (SKAT-O, R package) was performed.^27^ We separately analyzed the burden of all rare, nonsynonymous and functional variants (nonsynonymous, stop/frameshift and splicing) and loss-of-function variants. Lastly, we analyzed variants with a Combined Annotation Dependent Depletion (CADD) score of ≥ 20, representing the top 1% of potentially deleterious variants. For each of the analyses, we performed a meta-analysis between the cohorts using metaSKAT package,^28^ adjusting for sex, age and ethnicity. We applied false discovery rate (FDR) correction to all p-values. All the code used in the current study is available at https://github.com/gan-orlab/ARSA

## Results

### Rare functional and loss-of-function *ARSA* variants are associated with Parkinson’s disease

The average coverage across all four cohorts sequenced at McGill was >714X with >98% of the nucleotides covered at >30x (detailed in Supplementary Table 3). We identified a total of 96 rare variants across all cohorts sequenced at McGill (Supplementary Table 4) and 113 rare variants in AMP-PD and UKBB cohorts (Supplementary Table 5).

Burden analyses, using SKAT-O, demonstrated an association of functional variants with PD in four out of six cohorts (McGill, P=0.023, Columbia, P=0.037, Pavlov, P=0.022 and UKBB, P=0.009) and in the meta-analysis (P=0.042; Table 1; Supplementary Table 6). We also found an association between rare loss-of-function variants in the UKBB cohort (P=0.005) and in the meta-analysis (P=0.049). However, these results should be interpreted with caution as only a single loss-of-function variant was reported in the Columbia cohort, two in Pavlov and Human brain cohort, three variants in UKBB and two in AMP-PD (Supplementary Tables 4-5) and none of the associations survived FDR correction (Supplementary Table 6).

We found associations between all rare variants and PD in the McGill cohort (P=0.011), Columbia cohort (P=0.005), Pavlov and Human brain institute (P=0.019) and in the UKBB cohort (P=0.009). However, there was no association in the meta-analysis (Table 1; Supplementary Table 4). Variants with CADD scores ≥20 were associated with PD in the Columbia cohort (P=0.009), whereas no association was found in the other cohorts and in the meta-analysis. Similarly, all rare nonsynonymous variants in *ARSA* were associated with PD in the McGill cohort (P=0.032) but not in the other cohorts. We did not find the p.L300S *ARSA* variant, which was previously reported as pathogenic in PD,^29^ yet we found the likely pathogenic (based on Varsome annotation) p.L300V variant in two cases and one control in our analysis (Supplementary Tables 5-6).

### Evidence for association of the rare *ARSA* p.E384K in two families among Parkinson’s disease patients

**Figure 2.**
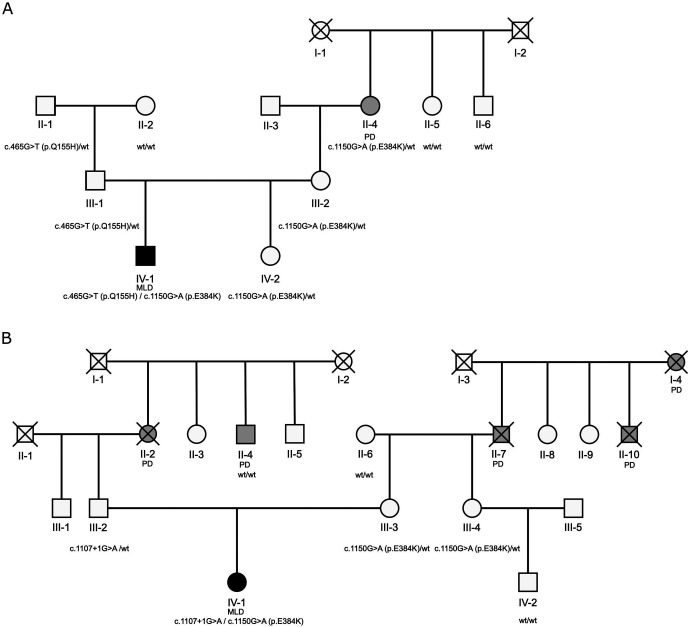
Family trees of two families with Metachromatic leukodystrophy and Parkinson’s disease in history. Square – male; circle – female; open symbol – healthy; grey– Parkinson’s disease; filled black symbol – Metachromatic leukodystrophy; PD – Parkinson’s disease; MLD – Metachromatic leukodystrophy; crossed line – deceased subject; wt – wild-type.

We describe here two families with history of MLD and PD. In the first family (Figure 2A), the proband is a patient with MLD with compound heterozygous nonsynonymous variants, p.Q155H and p.E384K. The maternal grandmother of the proband (Figure 2A, II-4), who is a carrier of p.E384K, has PD. The patient had early PD onset (<50 years). Other healthy relatives in this maternal generation (II) were wildtype for this variant. In the second family, the proband is a MLD patient who has compound heterozygous mutations, c.1107+1G>A and p.E384K. There were five PD patients in this family from both the paternal and maternal sides (Figure 2B). On the paternal side, there were two PD patients, one was deceased, and one was not a carrier of the pathogenic variant c.1107+1G>A. On the maternal side, there were three PD patients, all deceased. The maternal grandmother was wildtype to this variant. Therefore, the grandfather who was a PD patient was likely a carrier of p.E384K.

## Discussion

In the current study, we report a possible association between rare functional and loss-of-function *ARSA* variants and PD. In four of our cohorts, we also identified a possible association between all rare and nonsynonymous variants and PD. We also found a potential segregation of a pathogenic variant, p.E384K, with PD in 2 families with family history of PD and MLD, albeit we could not confirm this for all affected family members as some of the family members with PD have passed away. The negative results previously reported for rare *ARSA* variants in PD could be attributed to sample size or ethnicity (Supplementary Table 7).^12-14^ Although the associations described in the present study do not survive correction for multiple comparisons, the fact that there were many nominal associations in independent cohorts may suggest that these associations are real.

A recent large scale burden analysis found an association between rare *ARSA* loss-of-function variants and PD.^10^ While a study from China did not find a statistically significant burden of rare *ARSA* variants in PD,^30^ they reported higher prevalence of loss-of-function variants in late-onset PD (0.25% in PD vs 0% in controls),^30^ which is in line with our results. However, our results should be interpreted with caution as none of our associations survived FDR correction and we only discovered a few carriers of private loss-of-function variants across all six cohorts. A recent study from Japan suggested that the *ARSA* p.L300S mutation was likely pathogenic in PD due to co-segregation within a family with two PD patients.^29^ We did not find this specific variant in our study. However, it is possible that the variant p.E384K could be associated with PD based on the data we gathered from two families with MLD and PD.

The enzyme encoded by *ARSA*, arylsulfatase A, has an important role in the lysosomal ceramide metabolism pathway. Galactosylceramide is hydrolyzed from sulfatides by arylsulfatase A, which is then further hydrolyzed to ceramide by galactosylceramidase,^31^ encoded by the putative PD gene *GALC*.^1^ Another PD gene, *GBA1*,^1, 32^ also plays an important role in ceramide metabolism, by hydrolyzing glucosylceramide to ceramide (Figure 1). *ARSA* is also important for myelin metabolism.^33^ Several studies suggested a link between *ARSA* and alpha-synuclein accumulation. Alpha-synuclein depositions were found in glial cells and microglia of MLD patient,^34^ and in *ARSA* knockout cells, the authors reported increased alpha-synuclein accumulation, secretion and propagation.^11^ The activity of ARSA was reported to be low in the subset of patients with parkinsonism.^35^ Moreover, plasma ARSA level was reported to be higher in early PD as compared to controls or late PD, suggesting possible compensatory mechanism.^36^ Reduced level of sulfatides, substrate of ARSA, was reported in frontal cortex of PD patients.^37^ Therefore, there is biochemical, functional, and genetic evidence for the involvement of *ARSA* in neurodegeneration and potentially PD, further emphasizing the importance of the lysosomal ceramide metabolism pathway in PD (Figure 2). The link between *ARSA* and PD is not as strong as between *GBA1* and PD and only evident in large scale burden analysis (Supplementary Table 7). Potentially, it could be due to rarity of *ARSA* variants that associated with PD and could depend on the ethnicity.

Our study has several limitations. In some of our cohorts, patients and controls were not matched for sex and age, which was therefore adjusted in the statistical analysis. Quality control procedures were performed independently for targeted sequencing, whole exome and whole genome sequencing data using different thresholds for depth of coverage and quality control. This could potentially lead to discrepancy in enrichment in variants between different cohorts. Another limitation of our study is the inclusion of mainly individuals of European ancestry.

To conclude, rare functional and loss of function *ARSA* variants may be associated with PD, yet the results here cannot be considered as conclusive. Further replications in other cohorts are required to confirm our findings along with additional functional studies to understand the potential mechanism.

## Supplementary Material

Supplement 1 Supplementary Table 1 Study population
Supplementary Table 2 Detailed information on the *ARSA* molecular inversion probes
Supplementary Table 3 Coverage details for *ARSA*
Supplementary Table 4 Rare *ARSA* variants for cohorts sequenced at McGill
Supplementary Table 5 Rare *ARSA* variants for UKBB and AMP-PD cohorts
Supplementary Table 6 Burden analysis
Supplementary Table 7 Previous rare variants analysis of *ARSA* in PD

## Figures and Tables

**Table 1.** Burden analysis of rare *ARSA* variants

Cohort	N cases	N controls	All rare variants, P	All non- synonymous variants, P	Functional variants, P	Loss of function, P	CADD > 20, P
Columbia cohort	917	486	0.005	0.060	0.037	0.313	0.009
Sheba cohort	683	553	0.195	0.745	0.095	-	0.664
McGill cohort	761	549	0.011	0.032	0.023	-	0.081
Pavlov and Human brain cohort	497	401	0.019	0.106	0.022	0.467	0.082
UKBB	602	15,000	0.009	0.686	0.009	0.005	0.539
AMP-PD	2,341	3,486	0.820	0.673	0.602	0.107	0.705
Meta-analysis of all cohorts	5,801	20,475	0.826	0.420	0.042	0.049	0.431

N, number; P, p value; UKBB, UK biobank; AMP-PD, Accelerating Medicines Partnership – Parkinson Disease; CADD, Combined Annotation Dependent Depletion score.

p-value presented without FDR adjustment, as no p-values survived after correction.
